# Supplementary material for: Increasing Prevalence of Plant‐Fungal Symbiosis Across Two Centuries of Environmental Change
Source: Glob Chang Biol. 2025 Oct 30;31(11):e70577. doi: 10.1111/gcb.70577 (PMC12573070; doi:10.1111/gcb.70577)
Supplement: Supplementary file 1 — Appendix S1: gcb70577‐sup‐0001‐AppendixS1.pdf. [file GCB-31-e70577-s001.pdf]

Appendix A

Appendix to "Increasing Prevalence of plant-fungal symbiosis across two centuries of environmental change"

Authors:

Joshua C. Fowler[1,2*]

Jacob Moutouama[1,3]

Tom E. X. Miller[1]

1. Rice University, Department of BioSciences, Houston, Texas 77006;

2. University of Miami, Department of Biology, Miami, Florida;

3. Department of Botany, University of British Columbia, 6270 University Blvd, Vancouver, BC V6T 1Z4

∗ Corresponding author; e-mail: jcf221@miami.edu.

Contents:

Appendix A includes: Figure A1 - Figure A15, Table A1, and Supporting Methods).

1091

Supplemental Figures

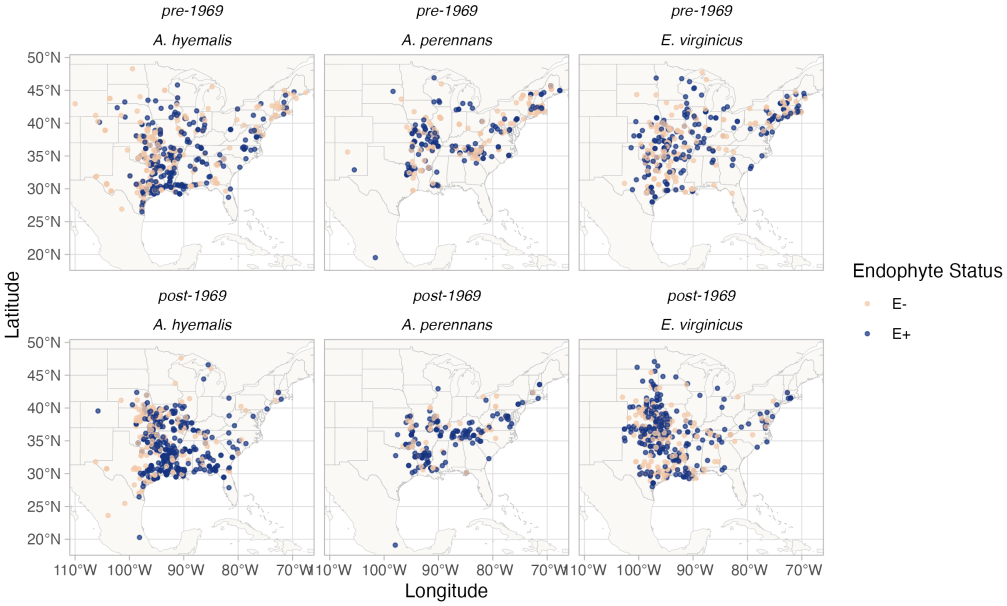

Figure A1: **Endophyte presence/absence in specimens of each host species.** Points show collection locations colored according to whether the specimen contained endophytes (E+; blue points) or did not contain endophytes (E-, tan points). To visualize temporal change, the data are faceted before and after the median year of collection. Map lines delineate study areas and do not necessarily depict accepted national boundaries.

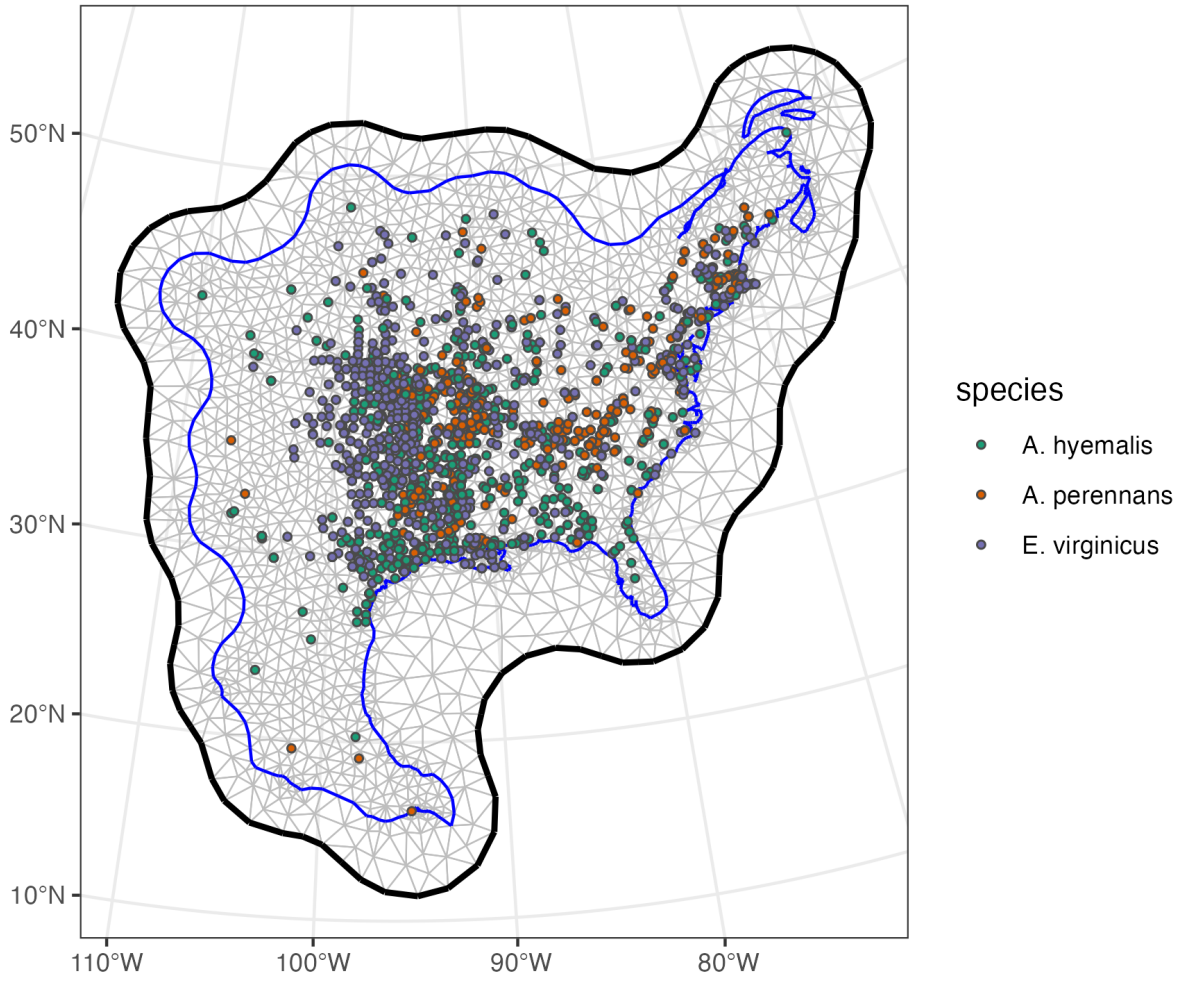

Figure A2: **Triangulation mesh used to estimate spatial dependence between data points**. Grey lines indicate edges of triangles used to define distances between observations. Colored points indicate locations of sampled herbarium specimens for each host species, and the blue line shows the convex hull and coastline used to define the edge of the mesh around the data points. The thick black line shows the convex hull defining a buffer space around the edge of the mesh to reduce the influence of edge effects on model estimates.

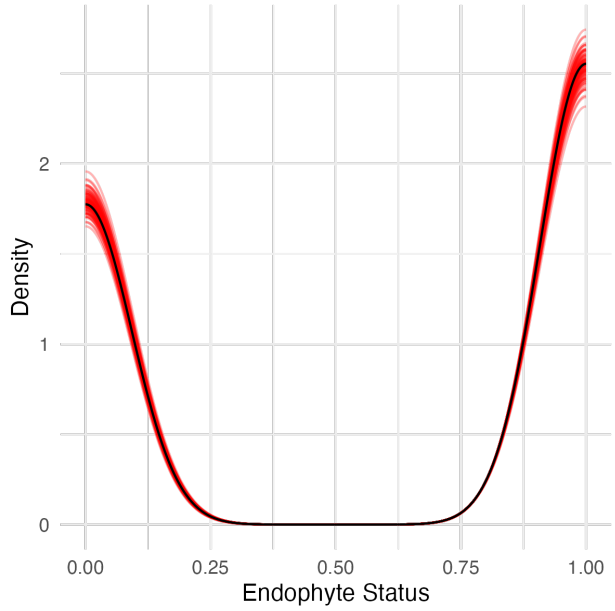

Figure A3: **Graphical posterior predictive check of the endophyte prevalence model fit.** Consistency between observed data and predicted values indicate that the fitted model accurately describes the data. Graph shows density curves for the observed data (black) along with 100 predicted datasets (red).

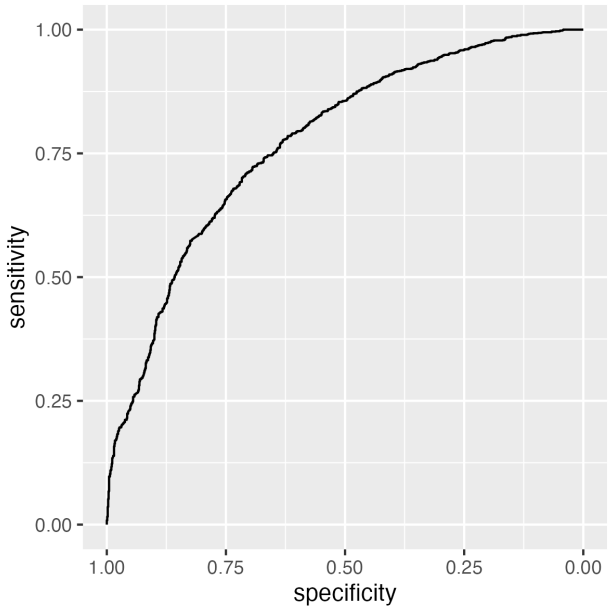

Figure A4: **ROC plot showing performance of the endophyte prevalence model in classifying observations according to endophyte status within the in-sample training data from herbarium collections.** The curves show adequate model performance for observed data. The AUC value is 0.79.

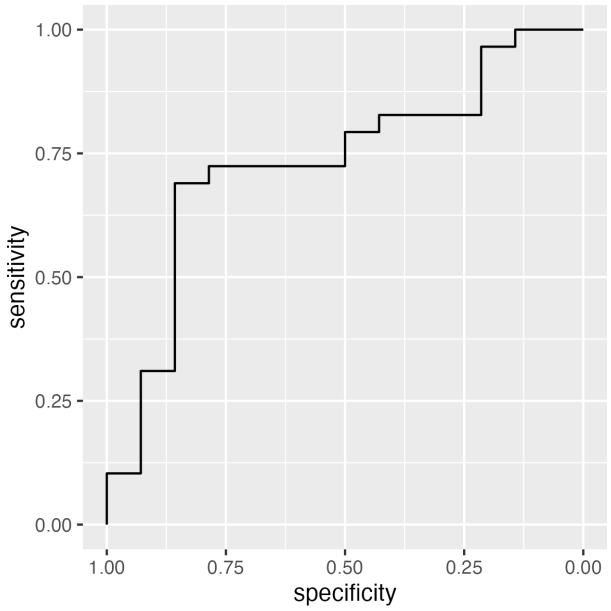

Figure A5: **ROC plot showing performance of the endophyte prevalence model in classifying observations according to endophyte status within the out-of-sample test data from contemporary surveys.** The curves show adequate model performance for test data. The AUC value is 0.77.

body of the text (Fig. A2), which we refer to as the "standard mesh", and with a mesh with smaller minimum vertices (finer mesh). Finer scale meshes increase computation time. For each of these meshes, we ran the model with a range of priors defining the spatial range of our spatial random effects: 342km (the prior used for presented results), as well as ranges five times smaller (68 km) and five times larger (1714 km). We found generally that these choices did not alter the direction of model predictions, but did influence the associated uncertainty and magnitude of some effects.

For overall temporal trends, we found that models with differing priors predicted consistently positive relationships over time (Fig. A16).
